# Enrichment in Different Health Components of Barley Flour Using Twin-Screw Extrusion Technology to Support Nutritionally Balanced Diets

**DOI:** 10.3389/fnut.2021.823148

**Published:** 2022-01-27

**Authors:** Sneh Punia Bangar, Kawaljit Singh Sandhu, Monica Trif, Alexandru Rusu, Ioana Delia Pop, Manoj Kumar

**Affiliations:** ^1^Department of Food, Nutrition and Packaging Sciences, Clemson University, Clemson, SC, United States; ^2^Department of Food Science and Technology, Maharaja Ranjit Singh Punjab Technical University, Bathinda, India; ^3^Food Research Department, Centre for Innovative Process Engineering (CENTIV) GmbH, Syke, Germany; ^4^Department of Food Science, Life Science Institute, University of Agricultural Sciences and Veterinary Medicine Cluj-Napoca, Cluj-Napoca, Romania; ^5^Department of Exact Sciences, Horticulture Faculty, University of Agricultural Sciences and Veterinary Medicine Cluj-Napoca, Cluj-Napoca, Romania; ^6^Chemical and Biochemical Processing Division, ICAR – Central Institute for Research on Cotton Technology, Mumbai, India

**Keywords:** barley, extrusion, resistant starch, phenolic, antioxidants

## Abstract

Due to its good dietary role, barley has attracted a growing amount of interest for the manufacture of functional foods in recent years. In barley, a number of bioactive components, including as phenolic compounds, have been discovered, and barley extrudates could be used to formulate various processed foods, including ready-to-eat cereals, baby, and pet foods and support nutritionally balanced diets. This study was conducted to investigate the effect of extrusion processing on resistant starch (RS), glycemic index (GI), and antioxidant compounds of barley flour. The L^*^ and Δ*E* values of barley flours decreased significantly (*p* < 0.05) after extrusion is done at 150 and 180°C. The a^*^ and b^*^ values, however, increased after extrusion. Extrusion increased antioxidant activity (AOA), metal chelating activity (MCA), and ABTS^+^ scavenging activity, whereas total phenolic content (TPC) and total flavonoids content (TFC) decreased. Barley extrudates at 150 and 180°C showed decreased TPC by 16.4–34.2% and 23.4–38.1%. Moreover, improved RS and reduced GI values were recorded for barley extrudates as compared to barley non-extrudates. Therefore, extrusion of barley could be an alternative to produce pregelatinized barley flour with improved RS low GI values and improved antioxidant potential.

## Introduction

Barley (*Hordeum vulgare* L.) is a grass family member and an ancient functional cereal crop grown in temperate climates worldwide. Globally, almost 51 million hectares land is cultivated for barley with a total production of 159 million tons (1). According to current knowledge, barley is the oldest cultivated grain in human history. It is mainly used for brewing beer. Barley mucilage has a positive effect on stomach diseases. Barley flour is not suitable for baking, as the bread tends to crack and crumble due to the fact it is not as rich in gluten as other grains. Gluten ensures that the bread rises in the baking process and keeps its shape.

Unlike in the past, most barley is currently processed into animal feed, it can be proven that the oldest cultivated cereal was also important for human nutrition in the past (2). Bread and baked goods could not be made from barley, as the fiber-rich grain has no baking properties, but it could be used to make a satisfying porridge. The advantage of barley bread, according to researchers, is its long shelf life and high fiber content. The researchers are particularly interested in the so-called beta-glucans, a soluble dietary fiber with cholesterol-regulating and blood sugar-lowering effects (3).

Nowadays, barley crop is receiving interest for preparing functional foods as a source of dietary constituents. Besides nutritional attributes, it is a good source of phytochemicals like phenols, flavonols, and tocopherols. Phenolic compounds include benzoic and cinnamic acid derivatives, proanthocyanidins, quinones, flavonols, flavones, flavanones, and amino phenolic compounds (4). The abundance of bioactive compounds confirms the barley's role in preventing many diseases and promoting health (5).

Thermal processing is an important part of food industries; to improve the quality and microbial safety of food products. During the past years, extrusion of food has evolved and now it has been a unique area of research. Extrusion processing is used to prepare expanded snacks, pet foods, porridge, and ready-to-eat cereal foods and modify starch (6). This thermal processing alters functional characteristics of starchy foods by pregelatinizing the starch and then followed by starch retrogradation, leading to retrograded resistant starch (RS). The RS formation is majorly dependent on the severity of extrusion parameters (7). The changes brought in the protein and starch contribute to the structure, texture, and mouthfeel of food products. Moreover, polyphenols undergo various changes during extrusion processing and decrease or increase depending on extrusion conditions (8).

The literature about extrusion processing on RS and glycemic index (GI) of extruded barley flour is still limited. Its nutritional profile makes barley an ideal candidate for developing a new extruded–expanded high fiber ready-to-eat snack food for example with health advantages. The research has been driving to see how extrusion processing factors affected system characteristics as well as physical qualities of barley flour extrudates (expansion, bulk density, texture, and color) until now (3, 9). Therefore, the present study's objective is to formulate barley flour extrudates and the effect of extrusion on RS, GI, and antioxidant properties of barley flour.

## Materials and Methods

Six cultivars (cv.BH-393, cv.BH-885, cv.BH-902, cv.BH-932, cv.DWR-52, and cv.PL-172) of barley were obtained from CCSHAU, Hisar, India. Cv. DWR-52 and cv.BH-885 were two-rowed and others barley cultivars were of six-rowed.

### De-husking, Milling, and Extrusion of Barley

Barley grains were de-husked using a rice polisher. Hulled barley grains were kept in the polisher chamber and run till the hull was removed entirely from the grains. Further, dehulled grains were ground in a Super Mill (Newport Scientific, Australia) and conditioned to 30% moisture content. The flours were packaged in polybags and equilibrated for 12 h. The extrusion of barley flours was carried out on a corotating twin-screw extruder (Clextral, Firminy, France). The screw and the diameter of 25 and 6 mm and (L/D) of 16 were adjusted with the feed rate of 20 kg/h and screw speed of 400 rpm. Barley grains were extruded at 150, and 180°C with barrel temperature of 50, 100, 125 and 150°C (for 150°C) and 50, 100, 140, and 180°C (for 180°C). An induction heating belt heated the terminal section, and the feeding area was cooled with running water.

### Hunter Color Characteristics

Hunter color of extruded barley was performed with a Hunter Colorimeter (Restan VA., USA) fitted with a sensor based on color scale [L^*^ = darkness (0) or lightness (100); a^*^ = greenness (–) or redness (+); b^*^ = blueness (–) or yellowness (+)]. L^*^a^*^b^*^ values were measured in triplicate, averaged, and recorded. Redness intensity (RI = a^*^/b^*^) was calculated for each sample, and correlations between color and sensory data were investigated. Calibration with a white standard plate, and its known L^*^a^*^b^*^ values, were completed prior to color testing.

### Resistant Starch and Glycemic Index

Resistant starch (RS) of barley flours was measured by adopting the method of Goñi et al. (10). The GI of barley flours was calculated following the process of Goñi et al. (10). In the process, *in vitro* study simulated the enzymatic digestion of starch. Hydrolysis index (HI) was measured by dividing the area under the hydrolysis curve of barley flours to the corresponding area of a reference sample i.e., white bread.

The GI was calculated using the following equation:

GI = 39.71 + 0.549 HI.

### Total Phenolic Content

Total phenolic content (TPC) of barley flour extrudates was evaluated using the Folin-Ciocalteu phenol reagent method (11). The quantity of TPC in barley extrudate extracts was determined using the standard calibration curve of gallic acid solution. Results were represented as μg gallic acid equivalents/g (μg g^−1^).

### Antioxidant Activity

Antioxidant activity (AOA) of barley grains was calculated by following the published method (12) using the methanolic DPPH solution. Methanol was used as a blank, and absorbance at the wavelength of 515 nm was read at 0 and 30 min.

Antioxidant activity was measured as percentage discoloration:

%AOA = (1 – (A of extracts_t = 30_/A of extracts_t = 0_)) × 100.

### Total Flavonoids Content

Total flavonoids content (TFC) was measured by adopting the method of Jia et al. (13). The quantity of TFC in barley extrudate extracts was determined using the standard calibration curve of standard catechin solution, and the results were expressed as μg of catechin equivalents (CE)/g.

### Metal Chelating Activity

Metal chelating activity (MCA) of extrudates was evaluated by adopting the method of Dinis et al. (14). Metal chelating activity of the samples for Fe^2+^was calculated by using this equation:

Iron (Fe^2+^) chelating activity (%) = {1 – (Abs of extracts/Abs of control)} × 100.

### ABTS (2,2′-azinobis 3-ethylbenzothiazoline-6-sulfonic Acid) Radical Cation Decolorization Assay

ABTS+ was calculated by following the already published method (15). The absorbance of extrudates was measured at a wavelength of 734 nm and compared with a standard curve using various concentrations of vitamin C. The result was expressed as vitamin C in μmol/g.

### Consumer Studies

Consumers studies of color attributes of barley flour were determined by six panelists (six assessors minimum requested by ISO 11035:2007) from Sensorial Lab, USAMV Cluj-Napoca, both males and females were represented in the panel. All of them have a long experience in product evaluation. Participants used a five-point Likert scale ranging from “dislike extremely” (1) to “like extremely” (7) for the color characteristics obtained by hunter color system.

In a further preliminary evaluation was investigated the role of color in consumers' acceptability and willingness-to-pay (WTP) was investigated. Consumers (*n* = 20) were randomly selected among students and staff of the USAMV Cluj-Napoca university (aged ranged 20–60). The outcome of this study is expected to provide support and guidance to current and future research on barley four extrudates.

Consumers were asked to complete a choice-based conjoint analysis to determine their WTP for barley flours obtained with different color characteristics indicated by hunter color system and health benefits indicated by results of analysis performed in this study.”

Price for barley flours was varied at three levels. Every choice question contained two options: (A) “I am willing to pay the price of a commercial barley flour” and (B) “I am willing to pay a higher price than a commercial barley flour, “plus a (C) “no buy” option.

### Data Analysis

The results generated during the analysis of barley flour extrudates was an average of three independent observations, which were further screened through ANOVA using the Minitab software ver. 16, and one-way ANOVA with *post-hoc* Tukey HSD Test. Results are presented in **Table 7**.

## Results and Discussion

### Color Parameters

The extruded barley cultivars were evaluated for their color characteristics by hunter color system (Table 1). The L^*^ and Δ*E* values of extruded samples reduced significantly (*p* < 0.05) when compared with control counterparts (data already published) and the values ranged from 83.6 to 88 and 84.6 to 89.4, respectively, for extrusion done at 150°C, and from 72.4 to 85.1 and 73.9 to 86.3, respectively, for extrusion done at 180°C. A reduction in L^*^ and Δ*E*-value could be due to the initiation and propagation of the Maillard reaction during extrusion resulting in producing brown pigments (16). It is evidenced that the L^*^ value reduced with increasing barrel temperature (B) while enhanced with increasing moisture content. High temperature and low moisture content favored the Maillard reaction (17). Maillard pigments contribute to the color, aroma, and flavor of food goods. The a^*^-value differed significantly (*p* < 0.05) among barley cultivars and increased after extrusion. It ranged from 1.01 to 1.79 and 1.34 to 2.03 for extrusion done at 150 and 180°C, respectively. The b^*^-value of flours from extruded barley cultivars also increased and differed significantly within the cultivars, with values of 9.3–12.6 (at 150°C) and 10.6–13.6 (at 180°C). An increase in redness and yellowness could also be attributed to Maillard products influenced by temperature, moisture content, reactant concentration, and reaction time (18).

**Table 1:** Hunter color characteristics of extruded (at 150 and 180°C) and non-extruded barley cultivars.

**Barley cultivars**	L*	a*	b*	**ΔE**	RI (a*/b*)
BH-393 150°C	84.$3_{\text{↓}7.5}^{\text{b}}$	1.0$1_{\text{↑}71.1}^{\text{a}}$	10.$18_{\text{↑}10}^{\text{b}}$	85.$3_{\text{↓}6.6}^{\text{b}}$	0.10
180°C	72.$4_{\text{↓}20.6}^{\text{l}}$	1.$63_{\text{↑}176}^{\text{m}}$	11.$36_{\text{↑}22.8}^{\text{m}}$	73.$9_{\text{↓}19.1}^{\text{l}}$	0.14
BH-932 150°C	86.$9_{\text{↓}2.9}^{\text{c}}$	1.$52_{\text{↑}10.1}^{\text{c}}$	12.$4_{\text{↑}9.2}^{\text{e}}$	87.$3_{\text{↓}2.8}^{\text{c}}$	0.12
180°C	82.$5_{\text{↓}7.8}^{\text{n}}$	1.$83_{\text{↑}32.6}^{\text{n}}$	13.$6_{\text{↑}19.8}^{∙}$	83.$9_{\text{↓}6.6}^{∙}$	0.13
BH-902 150°C	88.$0_{\text{↓}5}^{\text{e}}$	1.$13_{\text{↑}79.3}^{\text{a}}$	9.$3_{\text{↑}20}^{\text{a}}$	89.$4_{\text{↓}3.9}^{\text{f}}$	0.12
180°C	85.$1_{\text{↓}8.1}^{\text{p}}$	1.$34_{\text{↑}112}^{\text{l}}$	10.$6_{\text{↑}36.7}^{\text{l}}$	86.$3_{\text{↓}7.3}^{\text{q}}$	0.13
BH-885 150°C	87.$3_{\text{↓}3.4}^{\text{d}}$	1.$79_{\text{↑}58.4}^{\text{e}}$	12.$6_{\text{↑}9.3}^{\text{e}}$	88.$7_{\text{↓}2.3}^{\text{d}}$	0.14
180°C	80.$3_{\text{↓}11.1}^{\text{m}}$	2.$03_{\text{↑}79.6}^{∙}$	13.$1_{\text{↑}13.7}^{∙}$	82.$8_{\text{↓}8.8}^{\text{n}}$	0.15
DWR-52 150°C	83.$6_{\text{↓}7.7}^{\text{a}}$	1.$43_{\text{↑}38.8}^{\text{cd}}$	11.$47_{\text{↑}6.9}^{\text{d}}$	84.$6_{\text{↓}7.4}^{\text{a}}$	0.12
180°C	80.$1_{\text{↓}11.5}^{\text{m}}$	1.$69_{\text{↑}64}^{\text{m}}$	11.$91_{\text{↑}11.1}^{\text{n}}$	81.$2_{\text{↓}11.1}^{\text{m}}$	0.14
PL-172 150°C	87.$3_{\text{↓}2.1}^{\text{d}}$	1.$32_{\text{↑}22.2}^{\text{b}}$	10.$72_{\text{↑}9.2}^{\text{c}}$	88.$3_{\text{↓}1.9}^{\text{e}}$	0.12
180°C	84.$5_{\text{↓}5.2}^{∙}$	1.$63_{\text{↑}50.9}^{\text{m}}$	11.$3_{\text{↑}15.1}^{\text{m}}$	85.$3_{\text{↓}5.3}^{\text{p}}$	0.14

a–f superscripts are significantly (p <0.05) different within column for extrusion done at 150°C. l–q are significantly (p <0.05) different within column for extrusion done at 180°C. Subscripts denote the percentage increase (↑) and decrease (↓) from control samples for corresponding properties. L^*^ = darkness (0) or lightness (100); a^*^ = redness (+); b^*^ = yellowness (+), redness intensity (RI = a^*^/b^*^), total difference of color value (ΔE).

**Figure 1:**
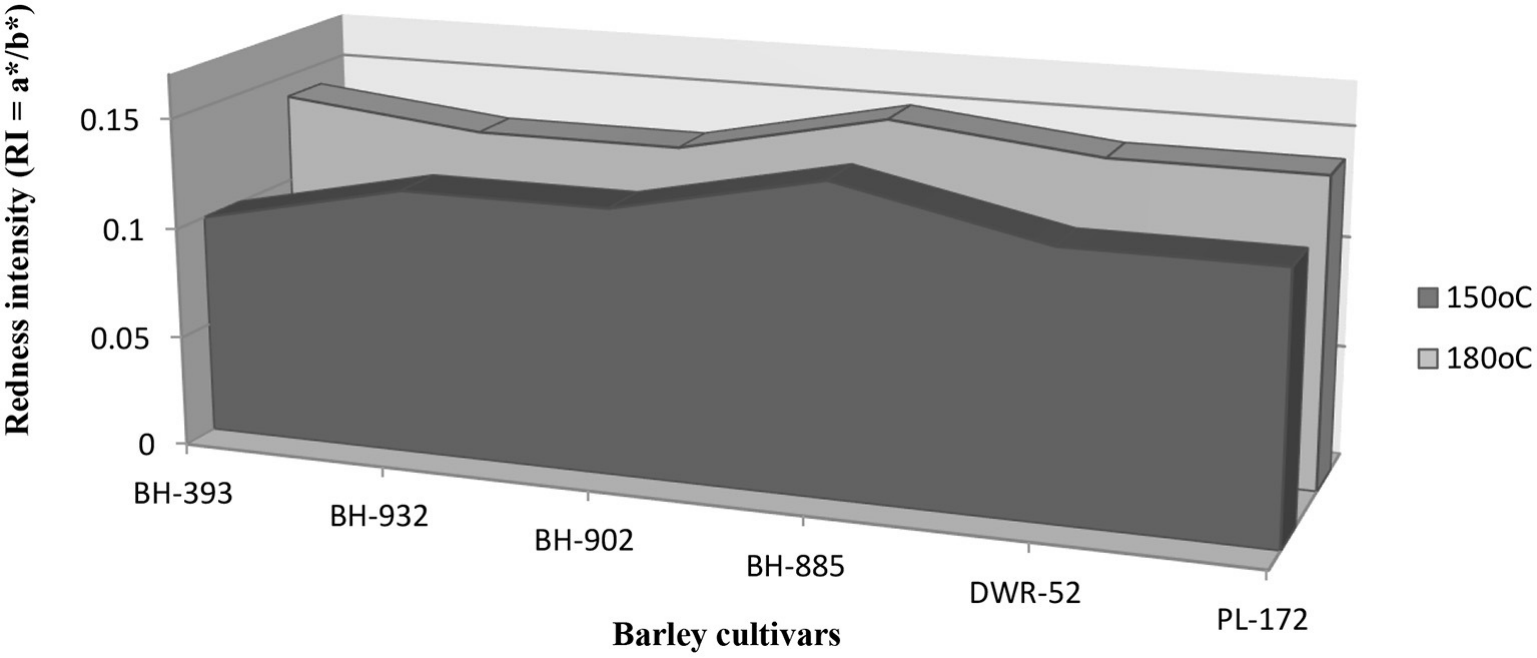
Graphic representation of redness intensity (RI = a*/b*) between barley cultivars.

Overall, RI increased from 150°C (range: 0.41–0.57) to 180°C (range: 0.51–0.60) (Table 1; Figure 1), and differences between cultivars are representative.

### Resistant Starch

Resistant starch in barley flour extrudates was calculated in the range of 1.7–2.78% at 150°C and 2.16–2.96% at 180°C compared to 1.33–2.2% recorded in barley flour non-extrudates (Table 2). Gulzar et al. (17) also recorded an enhancement of 4.91–6.83% in RS content of extruded rice flour. During extrusion, heat, and moisture content alter starch characteristics simultaneously, leading to interacting with non-starch components, thus supporting the retrograded starch production (17). Moisture behaves as a plasticizer for the starch retrogradation process, and at 30–60% moisture content, retrogradation is at its maximum (19). It is depicted from the results that there was a corresponding increase in RS content with increasing temperature and moisture. It is reported that the production of starch lipid complexes during the extrusion process, resulting in the formation of RS, which has the potential to lower starch digestibility (20). Formation of starch-lipid complexes can be identified by the V-type x-ray diffraction pattern. As a low-calorie component, RS became the interest of food enterprises and dietitians. It improves the food structure and shifts the consumption trend to healthy and nutritious food with nutrition and health care functions. Resistant starch could help in the prevention of increased blood sugar and avoid cancers and cardiovascular diseases (21).

**Table 2:** Resistant starch of extruded (at 150 and 180°C) and non-extruded barley flours.

**Barley cultivars**		**Resistant starch (RS) (%)**	
	**Non-extruded**	**150**°**C**	**180**°**C**
BH-393	1.52^b^	1.$87_{\text{↑}23}^{\text{b}}$	2.$26_{\text{↑}48.6}^{\text{b}}$
BH-932	1.56^b^	2.$08_{\text{↑}33.3}^{\text{c}}$	2.$56_{\text{↑}64.1}^{\text{c}}$
BH-902	1.99^cd^	2.$78_{\text{↑}39.6}^{\text{e}}$	2.$96_{\text{↑}48.7}^{\text{e}}$
BH-885	1.33^a^	1.$7_{\text{↑}27.8}^{\text{a}}$	2.$16_{\text{↑}62.4}^{\text{a}}$
DWR-52	2.2^d^	2.$34_{\text{↑}6.3}^{\text{d}}$	2.$82_{\text{↑}28.1}^{\text{d}}$
PL-172	1.93^c^	2.$15_{\text{↑}11.3}^{\text{cd}}$	2.$57_{\text{↑}33.1}^{\text{c}}$

*a–e superscripts are significantly (p <0.05) different within column for extrusion done at 150°C and 180°C. Subscripts denote the percentage increase (↑) and decrease (↓) from control samples for corresponding properties*.

### Glycemic Index

The GI of barley flour extrudates was found to be in the range of 20.6–23.3 at 150°C and 19.2–22.7% at 180°C and was significantly lower than recorded in barley flour non-extrudates (21.1–24.6%) (Table 3). Barley flour extrudates recorded a significant increase in RS with increased temperature, which likely reduced the GI-value. It is reported that the extent of decrease in GI-value is more pronounced at high moisture content. Extrusion of starchy foods at high moisture content leads to starch gelatinization and alteration of the native structure (17). Extrusion followed by drying results in the reorganization of the crystalline domain within the amorphous domain. This reorganization resulted in retrogradation results in a decrease in GI and an increase in RS content. These findings agree with previous studies of Hussain et al. (22), where an enhancement in RS with the reduced GI in preconditioned water chestnut flour was observed.

**Table 3:** Glycemic index of flours from extruded (at 150 and 180°C) and non-extruded barley flours.

**Barley cultivars**		**Resistant starch (RS) (%)**	
	**Non-extruded**	**150**°**C**	**180**°**C**
BH-393	24.6^e^	23.$2_{\text{↓}5.6}^{\text{d}}$	22.$4_{\text{↓}8.9}^{\text{d}}$
BH-932	23.8^cd^	22.$8_{\text{↓}4.3}^{\text{cd}}$	22.$7_{\text{↓}4.6}^{\text{d}}$
BH-902	22.6^b^	21.$9_{\text{↓}3.0}^{\text{bc}}$	20.$3_{\text{↓}10.1}^{\text{b}}$
BH-885	24.1^d^	22.$6_{\text{↓}6.2}^{\text{c}}$	22.$1_{\text{↓}8.2}^{\text{c}}$
DWR-52	21.2^a^	20.$6_{\text{↓}2.8}^{\text{a}}$	19.$2_{\text{↓}9.4}^{\text{a}}$
PL-172	23.3^c^	21.$2_{\text{↓}9.0}^{\text{b}}$	19.$3_{\text{↓}17.1}^{\text{a}}$

*a–e superscripts are significantly (p <0.05) different within column for extrusion done at 150°C and 180°C. Subscripts denote the percentage increase (↑) and decrease (↓) from control samples for corresponding properties*.

In the actual context of growing trends in high RS and low GI diets, can be concluded from this study that extrusion of barley at 150 and 180°C is a processing method of great importance in enhancing of RS and in the same time reducing the GI.

### Total Phenolic Content

Total phenolic content (TPC) of non-extruded barley flours ranged between 2,890 and 3,922 μg GAE/g. A decrease in TPC values was observed upon extrusion at 180 and 150°C temperature. Total phenolic content in barley extrudates at 180°C ranged from 1,788 to 2,879 μg GAE/g with the greatest and lowest for cv. BH-902 and cv. PL-172. In extrudates at 150°C, TPC differed significantly among barley cultivars and varied between 1,899 to 3,277 μg GAE/g (Table 4). Similar results were also observed on the TPC of sorghum extrudates (23). The authors observed a decrease of 11.8–20.0% in the free phenolic content of sorghum. However, Ramos-Enríquez et al. (24) evaluated the impact of extrusion processing on wheat bran at 140 and 180°C at 30% moisture content and observed an enhancement of bound TPC. In conclusion, the extrusion process either cause degradation and destruction of the molecular structure of heat-labile phenolic compounds or disintegrate the cell wall matrix and breaks covalent bonding of phenolics resulting in improved phenolic compounds (25–27). Besides, moisture content also affects phenolic content; at low moisture content, the shear force is higher, resulting in phenolics more prone to thermal degradation (28).

**Table 4:** Total phenolic content (TPC, μg GAE/g) of extruded (at 150 and 180°C) and non-extruded barley flours.

**Barley cultivars**	**Non-extruded**	**150**°**C**	**180**°**C**
BH-393	3256^d^	$2246_{\text{↓}31}^{\text{c}}$	$2013_{\text{↓}38.1}^{\text{c}}$
BH-932	3056^c^	$2348_{\text{↓}23.1}^{\text{d}}$	$2045_{\text{↓}33}^{\text{c}}$
BH-902	3761^e^	$3108_{\text{↓}17.3}^{\text{e}}$	$2879_{\text{↓}23.4}^{\text{e}}$
BH-885	3922^f^	$3277_{\text{↓}16.4}^{\text{f}}$	$2756_{\text{↓}29.7}^{\text{d}}$
DWR-52	2922^b^	$1935_{\text{↓}33.7}^{\text{b}}$	$1845_{\text{↓}36.8}^{\text{b}}$
PL-172	2890^a^	$1899_{\text{↓}34.2}^{\text{a}}$	$1788_{\text{↓}38.1}^{\text{a}}$

*a–e superscripts are significantly (p <0.05) different within column for extrusion done at 150°C and 180°C. Subscripts denote the percentage increase (↑) and decrease (↓) from control samples for corresponding properties*.

### Antioxidant Potentials

#### Determination of 1,1-diphenyl-2-picrylhydrazyl Radical Scavenging Activity

For the evaluation of the antioxidant potentialradical 1,1-diphenyl-2-picrylhydrazyl (DPPH) radical scavenging activity has been used to compare antioxidants, which quantifies the hydrogen donation potential of the compounds. The DPPH radical scavenging activity in the non-extruded barley flours ranged from 18.3 to 25.8% with cv.BH-885 and cv.PL-172 having the greatest and the lowest DPPH radical scavenging activity. The extrusion process led to a significant improvement in DPPH in all barley flour extrudates compared to non-extruded counterparts. An increase in DPPH could be due to the breaking of the covalent bond of the cell wall resulting in increased phenolic content and enhanced AOA (29), or the production of brown pigments (particularly melanoidins) due to the Maillard reaction (30). The production of browning pigments resulted in improved DPPH radical scavenging activity (16). 1,1-Diphenyl-2-picrylhydrazyl radical scavenging activity differed significantly among barley flour extruded at 150°C with 30% moisture. The DPPH radical scavenging activity ranged from 21.9% to 29.9%, with the greatest and lowest for cv. BH-902 and cv. PL-172, respectively. Similarly, at 180°C, the DPPH radical scavenging activity varied between 25.7 and 33.6% (Table 5). The BH-902 exhibited the greatest, and PL-172 exhibited the lowest DPPH radical scavenging activity. Delgado-Nieblas et al. (31) also reported the enhanced DPPH radical scavenging activity in breakfast cereals as the extrusion temperature was increased.

**Table 5:** Antioxidant potentials of extruded (at 150 and 180°C) and non-extruded barley flours.

**Barley cultivars**	**DPPH (%)**			**MCA (%)**			**ABTS**^**+**^ **(μmol/g)**		
	**Non-extruded**	**150**°**C**	**180**°**C**	**Non-extruded**	**150**°**C**	**180**°**C**	**Non-extruded**	**150**°**C**	**180**°**C**
BH-393	21.3^c^	23.$3_{\text{↑}9.3}^{\text{b}}$	27.$3_{\text{↑}28.1}^{\text{c}}$	44^c^	58.$4_{\text{↑}32.7}^{\text{d}}$	64.$2_{\text{↑}45.9}^{\text{d}}$	15.4^b^	17.$4_{12.9\text{↑}}^{\text{b}}$	19.$9_{29.2\text{↑}}^{\text{bc}}$
BH-932	20.5^bc^	24.$7_{\text{↑}20.4}^{\text{c}}$	26.$8_{\text{↑}30.7}^{\text{b}}$	38^b^	56.$6_{\text{↑}48.9}^{\text{c}}$	59.$2_{\text{↑}55.7}^{\text{b}}$	17.8^d^	20.$2_{13.4\text{↑}}^{\text{d}}$	21.$3_{19.6\text{↑}}^{\text{cd}}$
BH-902	24.9^d^	29.$9_{\text{↑}20}^{\text{d}}$	33.$6_{\text{↑}34.9}^{\text{e}}$	38^b^	55.$2_{\text{↑}45.2}^{\text{b}}$	60.$2_{\text{↑}58.4}^{\text{c}}$	16.8^c^	19.$2_{14.2\text{↑}}^{\text{c}}$	20.$8_{23.8\text{↑}}^{\text{c}}$
BH-885	25.8^e^	29.$2_{\text{↑}13.1}^{\text{d}}$	32.$7_{\text{↑}26.7}^{\text{d}}$	51^d^	61.$4_{\text{↑}20.3}^{\text{f}}$	69.$0_{\text{↑}35.2}^{\text{f}}$	15.1^b^	20.$6_{36.4\text{↑}}^{\text{d}}$	22.$2_{47\text{↑}}^{\text{d}}$
DWR-52	19.8^b^	24.$8_{\text{↑}25.2}^{\text{c}}$	27.$1_{\text{↑}36.8}^{\text{bc}}$	42^c^	59.$9_{\text{↑}42.6}^{\text{e}}$	65.$2_{\text{↑}55.2}^{\text{e}}$	13.2^a^	15.$6_{18.1\text{↑}}^{\text{a}}$	17.$1_{29.5\text{↑}}^{\text{a}}$
PL-172	18.3^a^	21.$9_{\text{↑}19.6}^{\text{a}}$	25.$7_{\text{↑}40.4}^{\text{a}}$	31^a^	47.$6_{\text{↑}53.5}^{\text{a}}$	53.$4_{\text{↑}72.2}^{\text{a}}$	15.3^b^	17.$5_{14.3\text{↑}}^{\text{b}}$	19.$1_{24.8\text{↑}}^{\text{b}}$

*a–e superscripts are significantly (p <0.05) different within column for extrusion done at 150°C and 180°C. Subscripts denote the percentage increase (↑) and decrease (↓) from control samples for corresponding properties*.

#### Metal Chelating Activity

Metal Chelating Activity (MCA %) is the most common method used for evaluating the antioxidant potential of substances. In the barley flours, MCA varied from 31 to 51% (Table 5). Upon extrusion, MCA increased in all the barley cultivars as compared to their non-extruded parts. Extrusion at 150°C with feed moisture of 30% improved the MCA, and the values ranged from 47.6 to 61.4%. cv. BH-885 showed the greatest and cv. PL-172 showed the lowest MCA. Sharma et al. (8) observed an increase in MCA of extruded barley up to 23%. They observed that the rise in MCA is due to novel substances (melanoidins) during thermal processing. However, with an increase in 150 to 180°C temperature with constant moisture content of 30%, improved MCA was observed. The greatest and the lowest MCA exhibited cv.BH-885 (69%) and cv.PL-172 (53.4%) (Table 5). Improved MCA upon increasing temperature and feed moisture could be due to the production of Maillard compounds with different concentrations. Moreover, produced Maillard compounds are melanoidins (high MW) and heterocyclic compounds (low MW), which are responsible for DPPH radical scavenging activity and MCA.

#### ABTS (2, 2′-azinobis 3-ethylbenzothiazoline-6-sulfonic Acid) Radical Cation Decolorization Assay

To measure the potential of antioxidants to quench the ABTS^+^ radicals, the reaction between antioxidants with ABTS^+^ occurs rapidly and can be assessed by following the reduced absorbance of samples at 734 nm. The AOA in the barley cultivars ranged from 13.2 to 17.8 μmol/g (Table 5). The extrusion cooking showed an increase in ABTS^+^ scavenging activity; however, a non-significant difference in barley extrudates at 150 and 180°C was observed. The ABTS^+^ activity of barley extrudates ranged from 15.6 to 20.6 μmol/g at 150°C and between 17.1 and 21.3 μmol/g at 180°C (Table 5). Results support the fact that thermal processing alters the antioxidant profile and produces more antioxidants contributing to AOA. Delgado-Nieblas et al. (31) reported an increase in ABTS^+^ scavenging activity of extruded cereal breakfast prepared from wheat and oat bran, yellow corn grits incorporated with naranjita pomace. It has been demonstrated that cereals have the potential to scavenge ABTS^+^ radicals, with potential effects in the reduction of lipid peroxidation (32).

### Total Flavonoids Content

Flavonoids have been associated to a variety of health benefits, including antioxidant potential benefits. Total flavonoids content (TFC) in the six barley cultivars varied from 1,968 to 2,198 μg CE/g. Upon extrusion, a significant (*p* < 0.05) reduction in the TFC was reported. The TFC of barley extrudates (150°C and 30% moisture content) different significantly among barley cultivars and ranged from 1,222 to 1,835 μg CE/g; BH-885 exhibited the greatest, and BH-902 showed the lowest TFC (Table 6). With increasing temperature from 150 to 180°C with the moisture content of 30%, a significant (*p* < 0.05) decrease was observed in TFC. Total flavonoids content ranged from 898 to 1,177 μg CE/g/g with the greatest and the lowest for BH-393 and PL-172. Zhang et al. (33) evaluated TFC of 0.992 mg rutin/g of buckwheat flour lowered by 33% after thermal treatment. A reduction in TFC could be due to the degradation of heat-sensitive flavonoids (34, 35).

**Table 6:** Total flavonoids content TFC (μg CE/g) of extruded (at 150 and 180°C) and non-extruded barley flours.

**Barley cultivars**	**Non-extruded**	**150**°**C**	**180**°**C**
BH-393	2011^b^	1,$602_{\text{↓}20.3}^{\text{d}}$	1,$177_{\text{↓}41.4}^{\text{d}}$
BH-932	2024^b^	1,$569_{\text{↓}22.4}^{\text{c}}$	1,$033_{\text{↓}48.9}^{\text{c}}$
BH-902	1988^ab^	1,$222_{\text{↓}38.5}^{\text{a}}$	$946_{\text{↓}52.4}^{\text{b}}$
BH-885	2198^c^	1,$835_{\text{↓}16.5}^{\text{e}}$	1,$339_{\text{↓}39}^{\text{e}}$
DWR-52	2002^b^	1,$546_{\text{↓}22.7}^{\text{c}}$	1,$033_{\text{↓}48.4}^{\text{c}}$
PL-172	1968^a^	1,$299_{\text{↓}33.9}^{\text{b}}$	$898_{\text{↓}54.3}^{\text{a}}$

*a–e superscripts are significantly (p <0.05) different within column for extrusion done at 150°C and 180°C. Subscripts denote the percentage increase (↑) and decrease (↓) from control samples for corresponding properties*.

Antioxidant supplements, according to evidence, do not work as effectively as antioxidants found naturally in foods. Antioxidant-rich foods may lower the risk of a variety of diseases (including certain cancers) (36). The present research is proving that barley extrudates are rich sources of antioxidants offering health benefits if integrated in a regularly diet from early life to be effective.

### Data Analysis

The results are presented in Table 7 below.

**Table 7:** One-way ANOVA with *post-hoc* Tukey HSD Test.

	**Tukey HSD Q statistic**	**Tukey HSD p-value**	**Tukey HSD inferfence**
**Resistant starch**
Non-extruded vs. 150°C	2.8533	0.1419605	NS
Non-extruded vs. 180°C	5.7305	0.0028074	^*^p < 0.01
150 vs. 180°C	2.8772	0.1379327	NS
**Glycemic index^+^**
Non-extruded vs. 150°C	2.2998	0.2655477	NS
Non-extruded vs. 180°C	4.2846	0.0216293	^**^p < 0.05
150 vs. 180°C	1.9848	0.3647908	NS
**TPC**
Non-extruded vs. 150	4.0349	0.030	^+^*p* < 0.05
Non-extruded vs. 180	5.2363	0.005	^++^*p* < 0.01
150 vs. 180°C	1.2014	0.669	NS
**DPHH**
Non-extruded vs. 150	2.9691	0.123	NS
Non-extruded vs. 180	5.4519	0.004	^±^*p* < 0.01
150 vs. 180°C	2.4828	0.217	NS
**MCA**
Non-extruded vs. 150	6.7487	0.001	^∅^*p* < 0.01
Non-extruded vs. 180	9.0266	0.001	^∅^*p* < 0.01
150 vs. 180°C	2.2779	0.271	NS
**ABTS^+^**
Non-extruded vs. 150	8.8792	0.001	^§^*p* < 0.01
Non-extruded vs. 180	7.7301	0.001	^§^*p* < 0.01
150 vs. 180°C	1.1491	0.690	NS
**TFC**
Non-extruded vs. 150	7.6732	0.001	^#^*p* < 0.01
Non-extruded vs. 180	14.1873	0.001	^#^*p* < 0.01
150 vs. 180°C	6.5141	0.001	^#^*p* < 0.01

*NS, not statistically significant*.

* *The p = 0.003 <0.05 corresponding to the F-stat = 8.20 of one-way ANOVA, suggesting that the samples are significantly different*.

** *The p = 0.02 <0.05 corresponding to the F-stat = 4.59 of one-way ANOVA, suggesting that the samples are significantly different*.

+ *The p = 0.005 <0.05 corresponding to the F-stat = 7.52 of one-way ANOVA, suggesting that the one or more sample are significantly different*.

± * The p = 0.005 <0.05 corresponding to the F-stat = 7.45 of one-way ANOVA, suggesting that the one or more sample are significantly different*.

∅ *The p = 0.000 <0.05 corresponding to the F-stat = 22.03 of one-way ANOVA, suggesting that the one or more sample are significantly different*.

§ *The p = 0.000 <0.05 corresponding to the F-stat = 23.31 of one-way ANOVA, suggesting that the one or more sample are significantly different*.

# *The p = 0.000 <0.05 corresponding to the F-stat = 50.43 of one-way ANOVA, suggesting that the one or more sample are significantly different*.

### Consumers Tests

**Figure 2:**
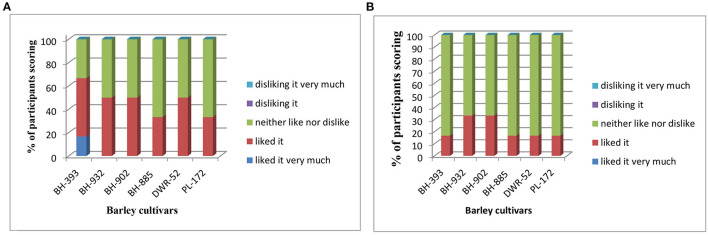
Results of the panelists tests (frequencies of scores for overall evaluation) for redness intensity (RI = a*/b*) between barley cultivars **(A)** extruded at 150°C, and **(B)** extruded at 180°C.

The panelists tests have showed some differences between the evaluation of at 150 and 180°C (Figure 2). Overall, all barley cultivars flours extruded scored between liked it or liked it very much almost same as neither like nor dislike. This result could be valuable for further applications. At 150°C flour extruded scored average 40.47% of panelists, liked it or liked it very much, compared to 52.78% of panelists neither like nor dislike. At 180°C flour extruded scored average 22.27% of panelists, liked it only, compared to 77.73% of panelists neither like nor dislike.

**Figure 3:**
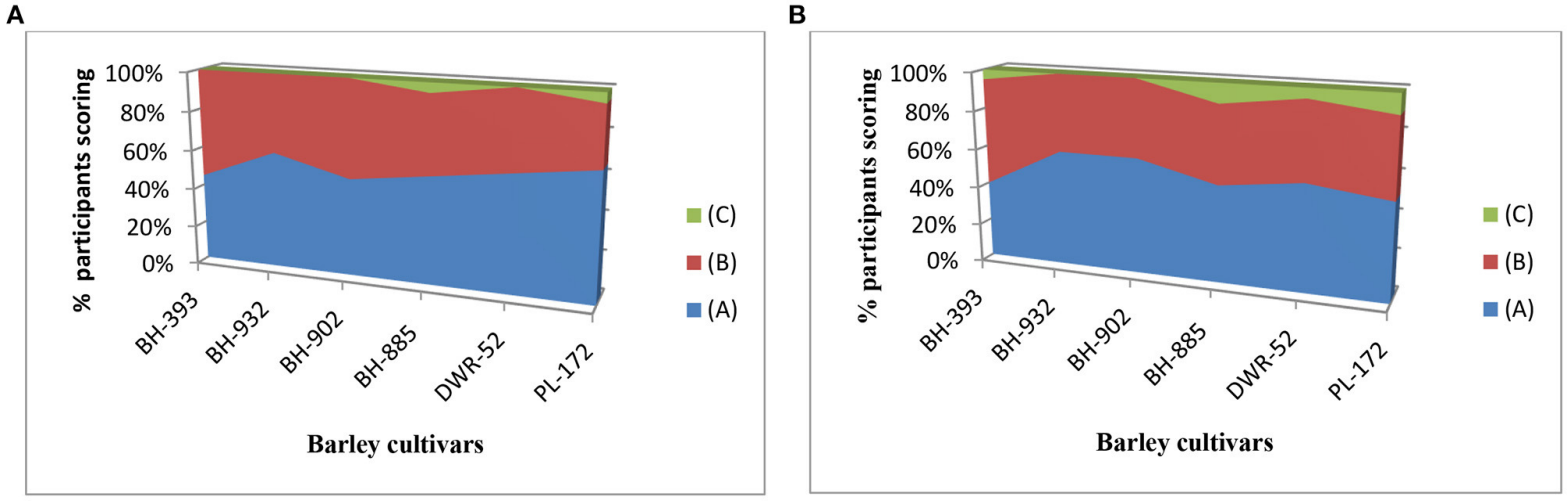
Results of the consumers studies willingness-to-pay (WTP) (frequencies of scores for overall evaluation) of barley cultivars **(A)** at 150°C, and **(B)**. **(A)** “I am willing to pay the price of a commercial barley flour, **(B)** “I am willing to pay a higher price than a commercial barley flour,” **(C)** “no buy.”

Furthermore, the choice-based conjoint analysis conducted for the barley cultivars at 150°C showed (Figure 3) that 55.83% consumers are willing to pay the price of a commercial barley flour and 42.5% are willing to pay a higher price than a commercial barley flour, relative to 1.66% not buying. Compared to choice-based conjoint analysis conducted for the barley cultivars at 180°C showed that 52.50% consumers are willing to pay the price of a commercial barley flour and same 42.5% are willing to pay a higher price than a commercial barley flour, relative to 5.0% not buying.

It is still relevant to mention that color and appearance of the food product is an important aspect considered by the consumers. If the food product deviates from the expected color, it can have an impact on consumer choices buy it or not. The discoloration of bread by barley in general having a dark gray color is one of the obstacles that prevent the use of barley in food products, and even when it is used in combination with other flour types, such as rice or wheat. It has been proven that the polyphenol content and level of polyphenol oxidase activity varies dramatically between different barley genotypes. The phenolic compounds are mainly found in the hull, testa, and aleurone of cereal grains, compared to barley.

Several solutions to reduce the discoloration of barley-containing foods have been proposed, such as heat-treatment in order to denature the polyphenol oxidase of barley flour resulting in less discoloration compared to unheated barley flour. However, these kind of processes result in a reduction of all polyphenolic content in the barley flour and decrease their health-promoting value.

Another alternative proposed is further research to identify which specific polyphenols have antioxidant properties (37), so that the development of barley genotypes that lack the discoloring polyphenols but have a high antioxidant polyphenol count can occur. Antioxidants have recently gained significant consideration, and one of the cereals containing most antioxidants is barley. People going to gym and train out, on the other hand, require bakery products to maintain a balanced diet. Looking at this trend the bakery and confectionary segments shall dominate the end-user category of global barley flour market between 2019 and 2029 (38).

## Conclusions

Extrusion has evolved as highly promising thermal processing method for producing foods and food ingredients. Extrusion processing positively affected the RS content significantly higher and GI significantly lower than recorded in barley flour non-extrudates of six barley cultivars tested. Barley subjected to high temperature formed brown pigments, which promote the antioxidant activities of barley flours. The results of present study demonstrated that the extrusion could be adopted to improve the barley flour quality, and therefore barley-rich functional foods with increased antioxidant activities can be develop and formulate, especially that the consumers' studies have shown the acceptance of the improved barley flour. The antioxidant benefits is well-recognized, and appropriate antioxidant nutrition plays an significant role in the development of functional food products tailored to certain life phases, lifestyles, or activity levels, as well as the support of food products functionality addressing specific health or wellbeing concerns.

## Code Availability Statement

MS office-2016 and ANOVA using the Minitab software ver. 16.

## Data Availability Statement

The original contributions presented in the study are included in the article/supplementary material, further inquiries can be directed to the corresponding author/s.

## Author Contributions

SP and AR drafted the manuscript. MK proofread the manuscript. All authors participated in the performing, generating and interpretation of results.

## Funding

Some work was supported by a grant from the Romanian National Authority for Scientific Research and Innovation, CNCS—UEFISCDI, project number PN-III-P2-2.1-PED-2019-1723 and PFE 17, within PNCDI III.

## Conflict of Interest

The authors declare that the research was conducted in the absence of any commercial or financial relationships that could be construed as a potential conflict of interest.

## Publisher's Note

All claims expressed in this article are solely those of the authors and do not necessarily represent those of their affiliated organizations, or those of the publisher, the editors and the reviewers. Any product that may be evaluated in this article, or claim that may be made by its manufacturer, is not guaranteed or endorsed by the publisher.
